# Supplementary material for: Hydration and Ion Pair Formation in Aqueous Lu3+- Solution
Source: Molecules. 2018 Dec 7;23(12):3237. doi: 10.3390/molecules23123237 (PMC6321123; doi:10.3390/molecules23123237)
Supplement: Supplementary file 1 [file molecules-23-03237-s001.pdf]

Supplementary Information

Table S1. Assignment and approximate description of the calculated vibrational modes using B3LYP/SDD of $[Lu(H_2O)_8]^{3+}$ and $[Lu(D_2O)_8]^{3+}$ applying the PC model to the contribution of the normal modes of the LuO_8 skeleton.

Peak position/ cm^{-1}		LuO_8 vibrations			Description of the corresponding Raman active Lu-OH_2 / LuOD_2 normal modes		
$[Lu(OH_2)_8]^{3+}$	$[Lu(OD_2)_8]^{3+}$	Mode	Symmetry	Activity	Restricted translation of H_2O/D_2O along Lu-O bond; angles O-Lu-O are fixed	Restricted rotation of H_2O/D_2O with resp. to Lu; Lu-O distance is fixed	(Small)H_2O/D_2O librational contributions
56.3	51.4	δ	e_2	R		x	x
117.3	106.8	δ	b_2	i.r.			
130.0	122.5	δ	b_1	n.a.			
132.1	120.4	δ	e_1	i.r.			
160.7	149.1	δ	e_1	i.r.			
163.9	150.5	δ	a_1	R		x	x
168.1	153.5	δ	e_3	R		x	x
176.3	186.2	δ	e_2	R		x	x
291.3	271.1	ν_{as}	e_2	R	x		x
198.3	285.6	ν_{as}	e_3	R	x		x
320.3	298.6	ν_{as}	b_2	i.r.			
337.3	314.6	ν_{as}	e_1	i.r.			
376.3	356.3	ν_s	a_1	R	x		

Remarks: The LuO_8 skeleton modes may be divided into modes with restricted translation or restricted rotation character of the water molecules. In the first case the O-Lu-O angles of the ligands, water molecules, remain fixed whereas in the second case the Lu-O distances remain fixed. Additionally, water molecules perform small librations as a whole (without H-O-H bending or O-H stretching). Small accompanying librations are necessary for reasons of symmetry because during the vibration the mass centre of the cluster is conserved. Only in the case of the total symmetric breathing vibration of the cluster the water molecules move along the Lu-O ligand bonds without any librational contribution or changes of the O-Lu-O angles.

Figures

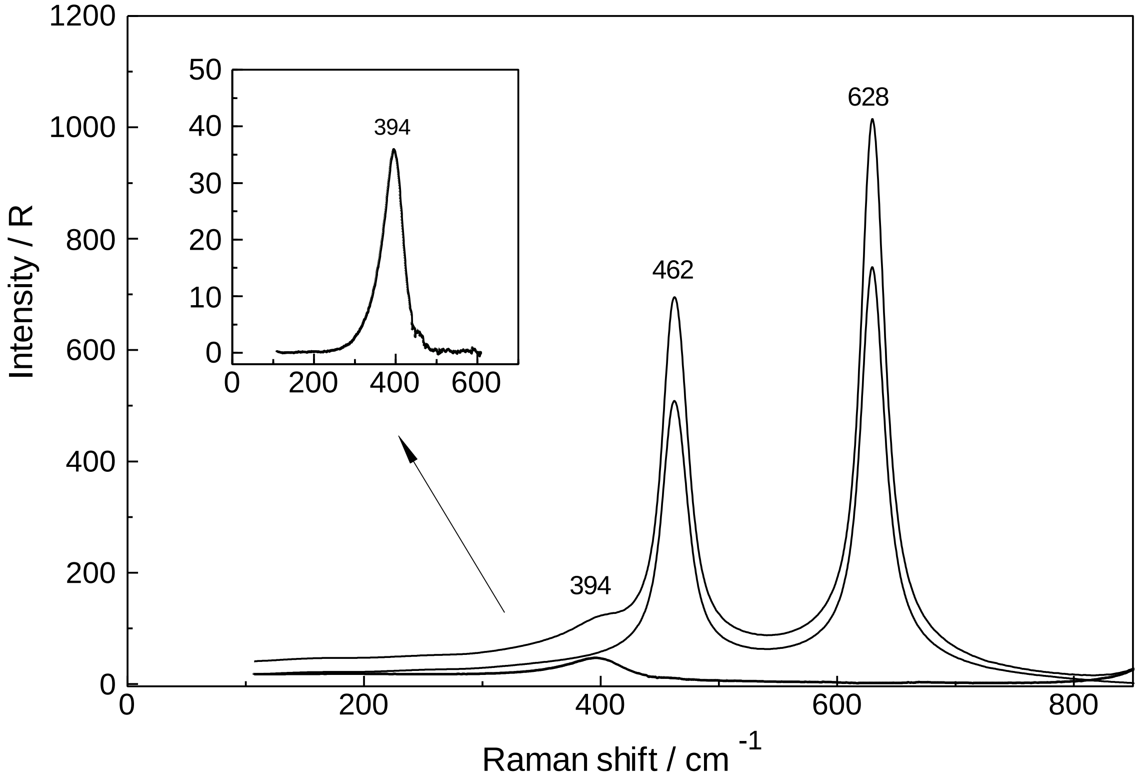

Figure S1. Raman scattering profiles in R-format (from top to bottom: polarized, depolarized and isotropic scattering profiles) of a 2.233 $molL^{-1}$ $Lu(ClO_4)_3$ solution (R_w = 19.01). The ClO_4^-(aq) deformation bands at 462 and 628 cm^{-1} are depolarized and therefore do not appear in the isotropic scattering. The inset shows ν_1 Lu-O stretching mode at 394 cm^{-1}, the breathing mode of the LuO_8 skeleton at the terahertz frequency range. The isotropic scattering profile is given.

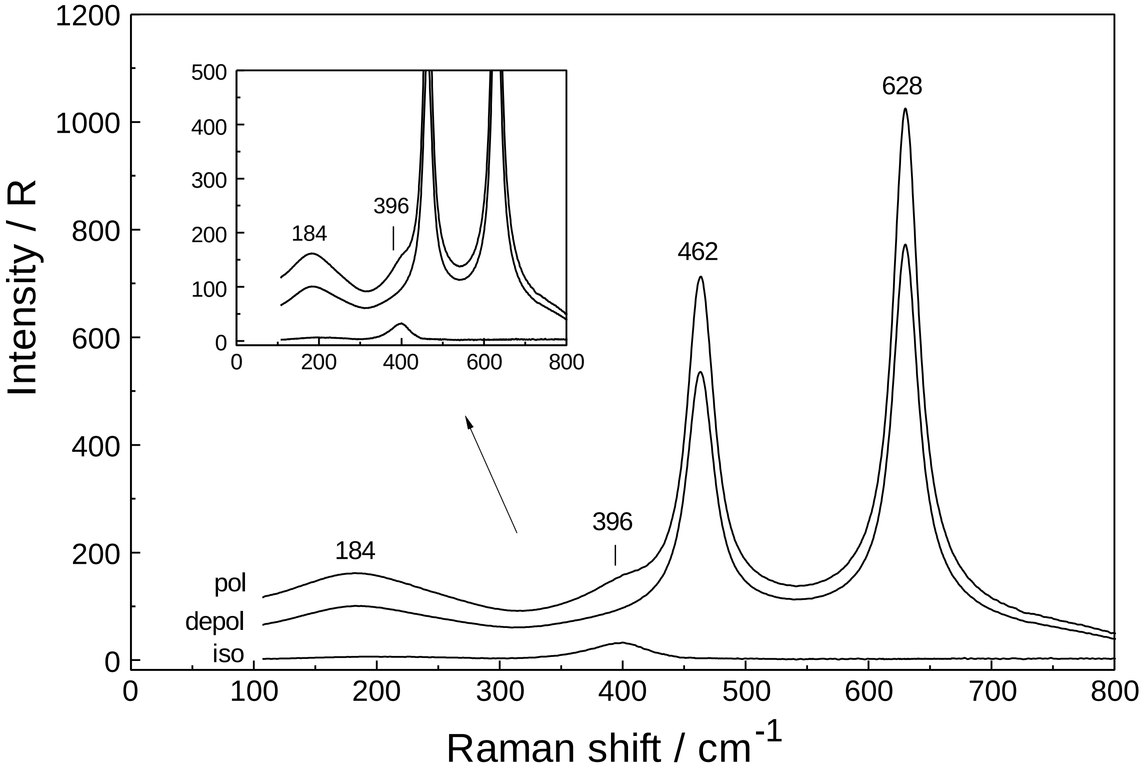

Figure S2. Raman scattering profiles in R-format (polarized, depolarized and isotropic scattering profiles) of a 0.556 molL^{-1} $Lu(ClO_4)_3$ solution (R_w = 92.37). The ClO_4^-(aq) deformation bands at 462 and 628 cm^{-1} are depolarized and therefore do not appear in the isotropic scattering. The inset shows symmetric stretching mode, ν_1 Lu-O, called the breathing mode of the LuO_8 skeleton at the terahertz frequency range at a larger scale. All scattering profiles are given.

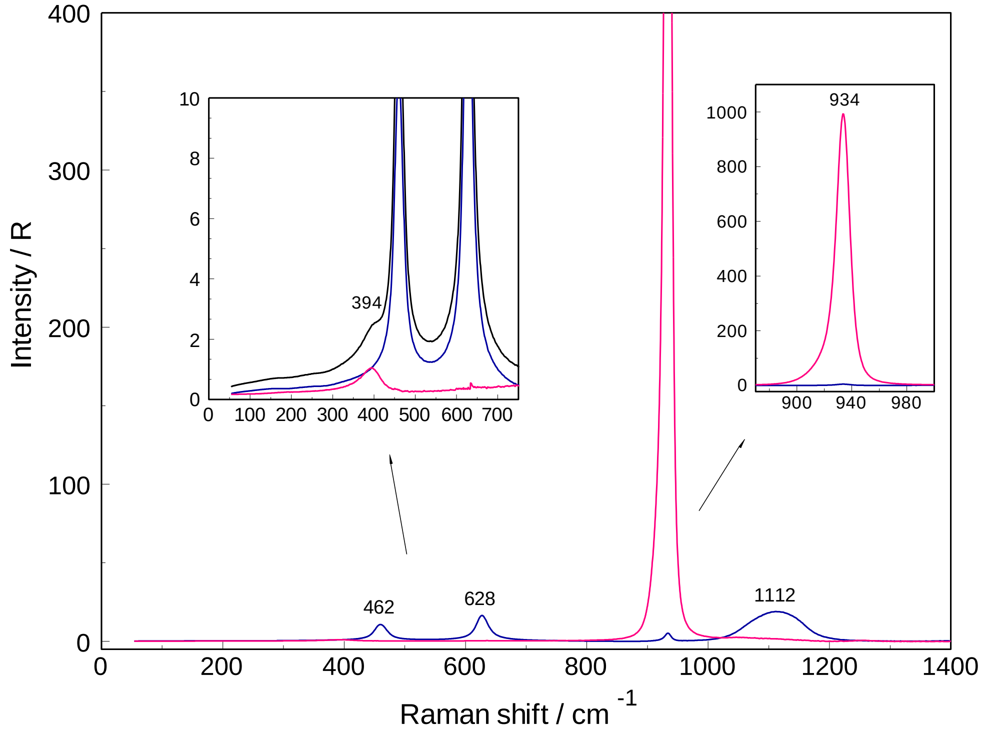

Figure S3A. Overview Raman scattering profiles in R-format (from top to bottom: polarized, depolarized and isotropic scattering profiles) of a 2.233 $molL^{-1}$ $Lu(ClO_4)_3$ solution (R_w = 19.01) from 50 – 1400 cm^{-1}. The ClO_4^-(aq) deformation bands at 462 and 628 cm^{-1} are depolarized and therefore do not appear in the isotropic scattering. The symmetric stretching mode, ν_1 of ClO_4^- (aq) appears at 934 cm^{-1} and the antisymmetric stretching mode ν_3 of ClO_4^-(aq) appears as a depolarized mode at 1112 $cm^{-1.}$ The inset at the left side shows ν_1 Lu-O stretching mode at 394 cm^{-1} the breathing mode of the LuO_8 skeleton at the terahertz frequency range at a larger scale. The inset at the right side shows the ν_1 ClO_4^- band at 934 cm^{-1} at its full scale.

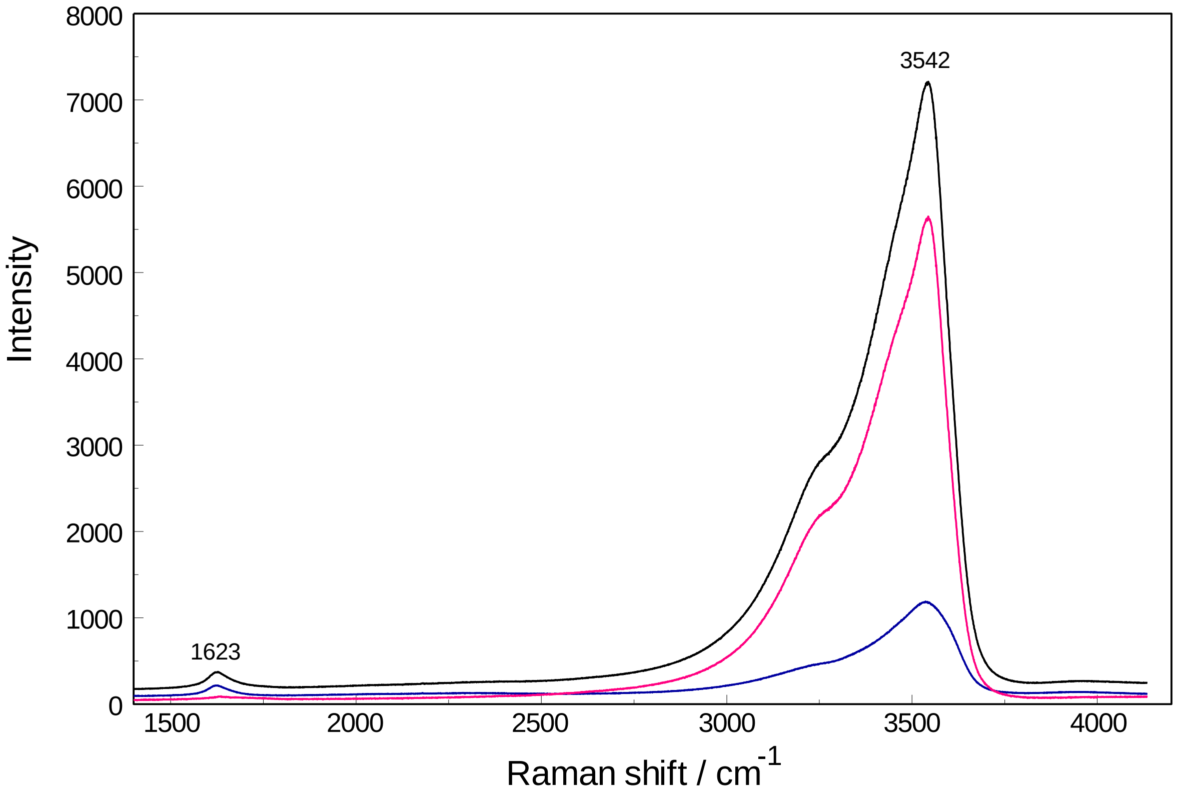

Figure S3. Raman profiles of the same 2.233 $molL^{-1}$ $Lu(ClO_4)_3$ solution in the wavenumber region from 1400 cm^{-1} to 4160 cm^{-1} (from top to bottom: polarized, depolarized and isotropic scattering profiles). The deformation mode of water appears at 1623 cm^{-1} and in the O-H stretching region the water band is modified by the typical ClO_4^- ··· H_2O mode of weakly hydrogen bonded O-H oscillators. The strong prominent band at 3542 cm^{-1} is due to the weakly bonded O-H…O units of water ··· ClO_4^-.

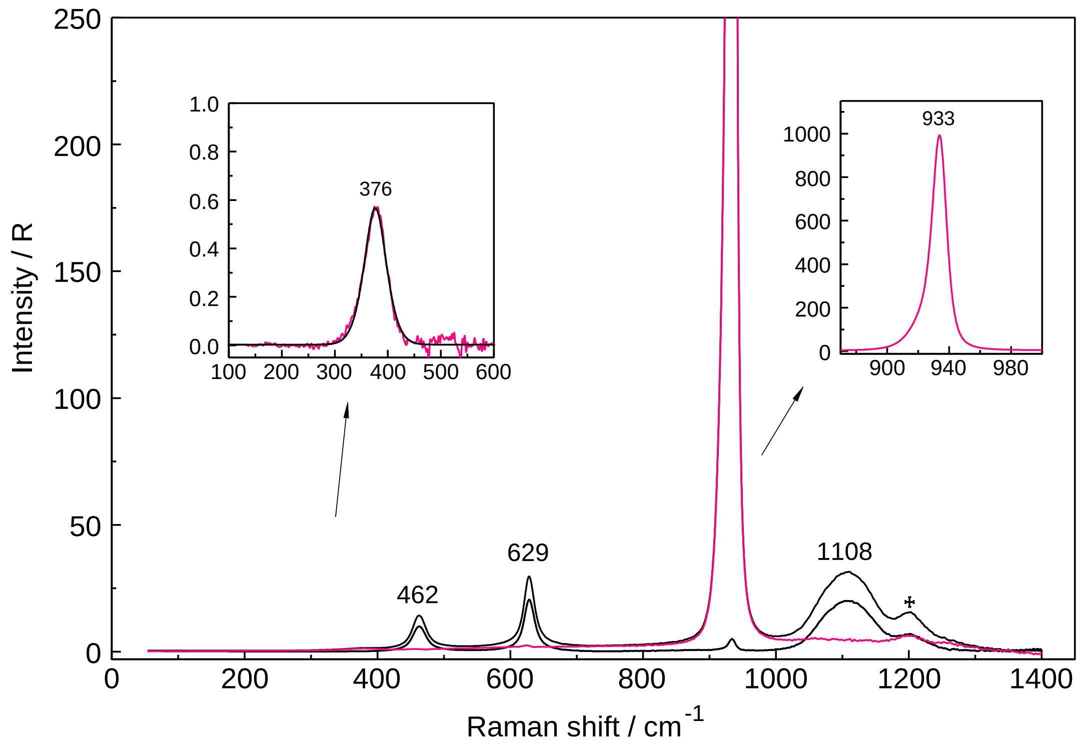

Figure S4. Raman spectrum in R-format (from top to bottom: polarized, depolarized and isotropic scattering profiles) of a 0.439 molL^{-1} $Lu(ClO_4)_3$ solution in D_2O. The ClO_4^- (D_2O) deformation bands at 462 and 629 cm^{-1} are depolarized and therefore do not appear in the isotropic scattering. The symmetric stretching mode, $\nu_1ClO_4^-$ (D_2O) appears at 933 cm^{-1} and the antisymmetric stretching mode, $\nu_3ClO_4^-$ (D_2O) at 1108 cm^{-1}. The latter mode is depolarized. The Lu-O breathing mode is shifted to 376.5 cm^{-1} compared to the mode in $Lu(ClO_4)_3$ in H_2O, where it appears at 396 cm^{-1}. The band marked with the symbol (+) at 1202 cm^{-1}, partially polarized, is due to the deformation mode of D_2O.

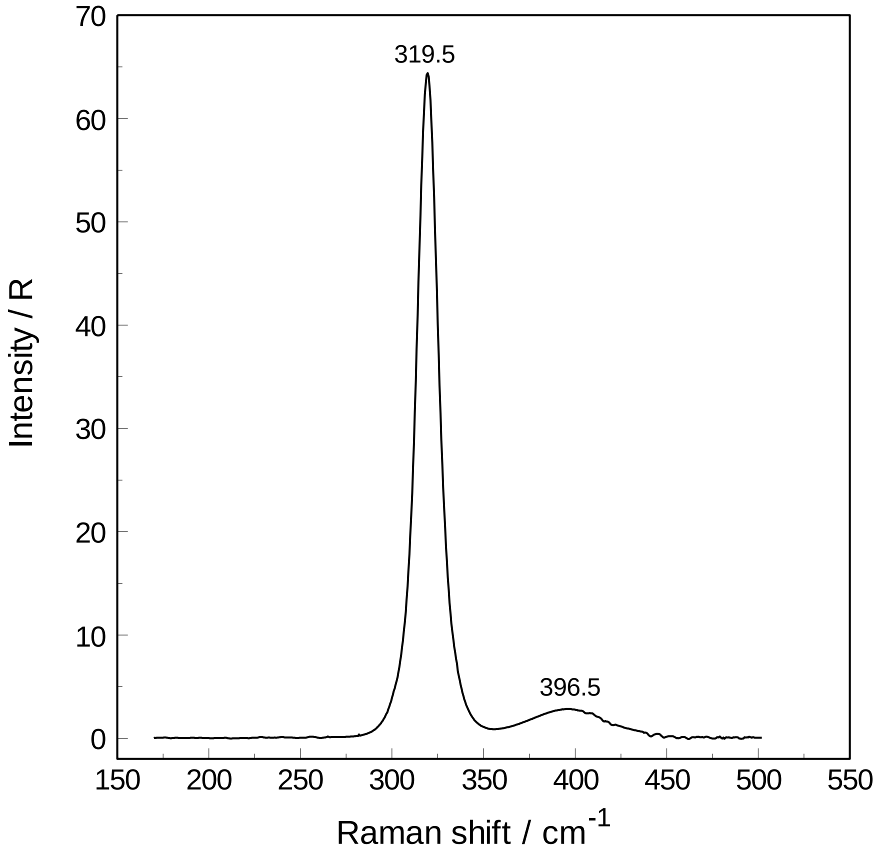

Figure S5. Isotropic Raman scattering profile (R-format) of a 1.05 molL^{-1} $Lu(CF_3SO_3)_3$ solution. Next to the strong band of triflate at 319.5 cm^{-1} appears a small and relatively broad band at 396.5 cm^{-1} which is due to the totally symmetric stretching mode of LuO_8.

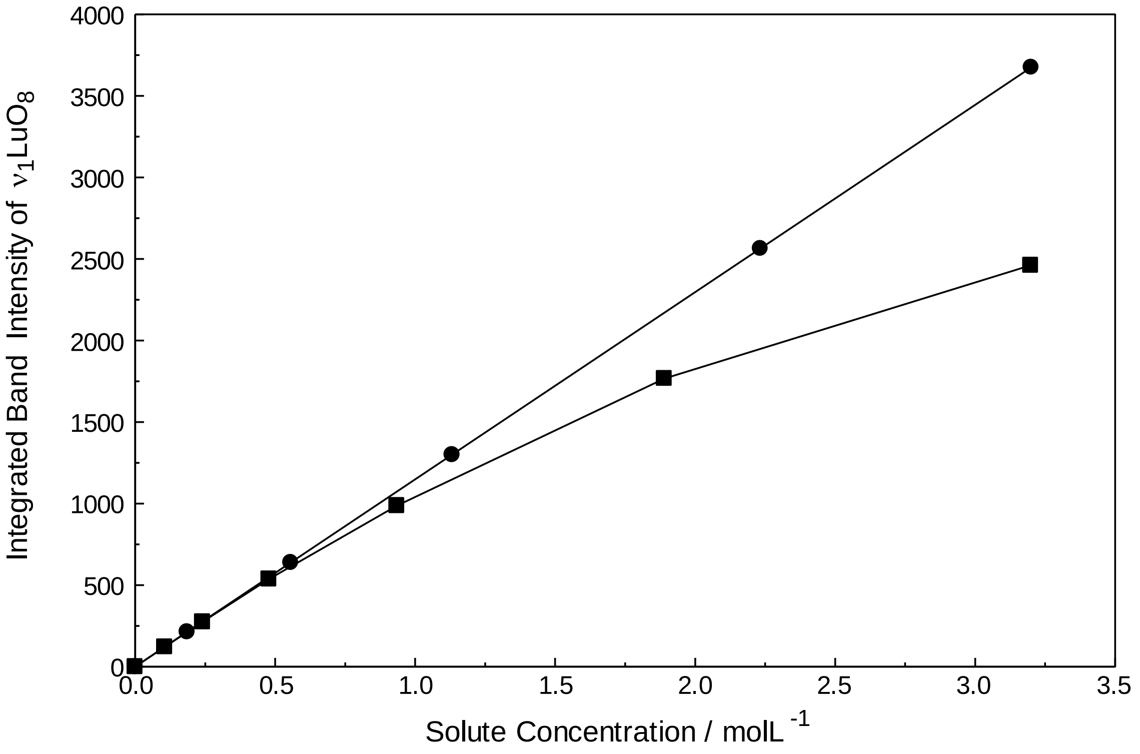

Figure S6. Linear regression line of the integrated band intensity I_{396} as a function of the $Lu(ClO_4)_3$ solution concentration (black circles); upper curve depicts: $I_{396}=1148.3*C_T$, the calibration curve. The lower curve shows the integrated band intensities in $LuCl_3$ solutions (black squares).
